# Trimethyloxonium-mediated methylation strategies for the rapid and simultaneous analysis of chlorinated phenols in various soils by electron impact gas chromatography–mass spectrometry

**DOI:** 10.1038/s41598-022-05463-w

**Published:** 2022-01-26

**Authors:** Carlos A. Valdez, Edmund P. Salazar, Roald N. Leif

**Affiliations:** 1 Forensic Science Center, Lawrence Livermore National Laboratory, 7000 East Ave., Livermore, CA 94550 USA; 2 Physical and Life Sciences Directorate, Lawrence Livermore National Laboratory, 7000 East Ave, Livermore, CA 94550 USA; 3 Nuclear and Chemical Sciences Division, L-090, Lawrence Livermore National Laboratory, 7000 East Ave, Livermore, CA 94550 USA

**Keywords:** Chemistry, Analytical chemistry

## Abstract

The efficient methylation of a panel of five industrial and environmentally-relevant chlorophenols (CPs) employing trimethyloxonium tetrafluoroborate (TMO) for their qualitative detection and identification by electron impact gas chromatography–mass spectrometry (EI-GC–MS) is presented. The protocol’s execution is simple and smoothly converts the phenols into their O-methylated counterparts conveniently at ambient temperature. The efficiency of two versions of the protocol was successfully tested in their ability to simultaneously derivatize five CPs (2-chlorophenol, 2,4-dichlorophenol, 2,4,6-trichlorophenol, pentachlorophenol and triclosan) in six distinct, separate soil matrices (Nebraska EPA standard soil, Virginia Type A soil, Ottawa sand, Baker sand, Silt and Georgia EPA standard soil) when present at low levels (~ 10 μgg^−1^). The first version involves the direct derivatization of the spiked soils with the methylating salt while the second one involves an initial soil extraction step of the CPs followed by methylation. The MDL values for each methylated CP were determined and lower values were found (4.1–13.2 ng^.^mL^−1^) for both sand matrices (Ottawa and Baker) as well as for the Georgia EPA standard soil, while larger values (8.2–21.8 ng^.^mL^−1^) were found for the Virginia Type soil, Nebraska EPA standard soil and Silt. The presented protocol offers a safer and more practical alternative to the universally employed diazomethane method and can be readily applicable to matrices other than soils. Furthermore, the protocols described herein may find applicability to the methylation of other analytes bearing acidic protons.

## Introduction

**Figure 1:**
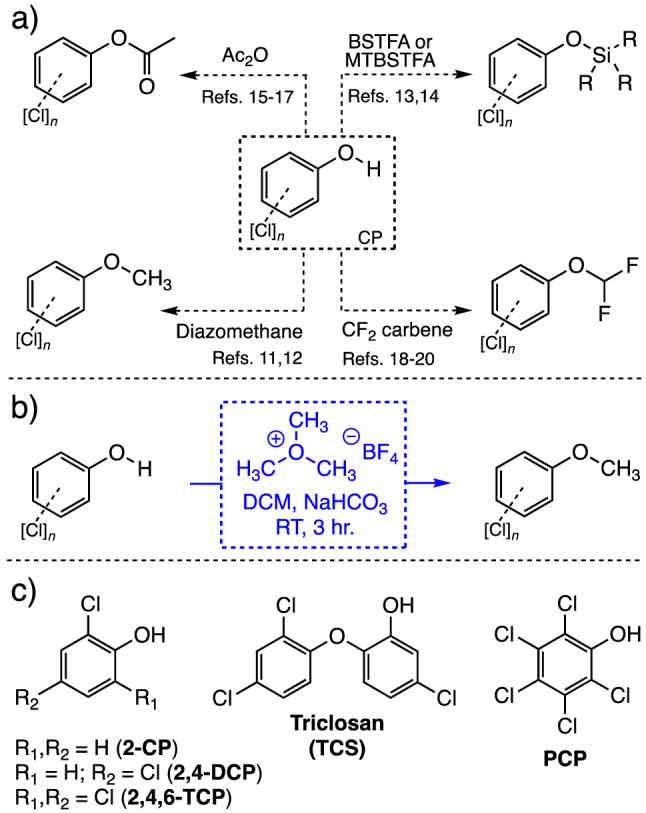
**(a)** Derivatization techniques available for the analysis of chlorinated phenols by GC–MS; **(b)** derivatization strategy involving trimethyloxonium tetrafluoroborate chemistry described in this work and **(c)** chemical structures of the five chlorinated phenols chosen for the proof-of-concept outlined in this paper.

Endocrine disrupting chemicals (EDCs) are compounds that have the ability to mimic hormones and thus, are able to interfere or cause disruption of various biological processes mediated by these important molecules^1^. Due to their potencies at low concentrations, EDCs have been a topic of mounting concern due to their potentially devastating impact on the ecosystem. Some of the well-recognized EDCs in the field of environmental and analytical chemistry include the pesticides, synthetic as well as natural estrogens like 17-β-ethinylestradiol and testosterone respectively, and the chlorinated phenols (CPs)^1,2^. Due to their wide employment in the manufacture of antiseptics, insecticides, wood preservatives^3^, the CPs along with other phenolic species such as bisphenols, have become present in virtually every ecosystem^4^. Government restriction policies across the globe have been forcefully established in an effort to diminish their impact on the environment^5^. As reports of their toxic effects started to accumulate, so did methods for their analysis and monitoring by various analytical means^6^ that have included detection methods based on chemiluminescence^7,8^, fluorescence^9^ and amperometric monitoring^10^. As expected, most of the analytical methods in the field have relied on the CP’s intrinsic UV absorption profiles (*e.g*. LC–MS coupled to UV detection)^11–15^ while others depend on the volatility of products arising from their derivatization (e.g. GC–MS)^16,17^. With regards to GC–MS analyses, methods for the derivatization of CPs abound in the literature and some of these include methylation using diazomethane^18,19^, silylation^20,21^, acetylation^22–24^ and more recently difluoromethylation^25–27^ (Fig. 1a).

With respect to methylation, diazomethane has been the gold standard when it comes to capping functional groups bearing acidic protons with the methyl group^28^. Conveniently, and aside from its unparalleled reactivity, diazomethane-mediated methylation of these materials does not yield by-products that can potentially act as interferences in the GC–MS analysis. Nevertheless, there are two major drawbacks in the employment of this reagent. The first one is diazomethane’s stability after its preparation, a characteristic that severely limits its effective use to a tight timeframe (i.e. 1–2 days). The second drawback is the explosive hazard associated with its preparation that has to occur constantly in an analytical chemistry laboratory^29^. To this end, other reagents that can carry out methylations and displaying better stability over diazomethane have been developed some of these include trialkylsilyldiazomethane^30,31^ and a set of alkylating reagents that are starting to find applications in the field of analytical chemistry, the trialkyloxonium salts such as trimethyloxonium tetrafluoroborate (TMO). TMO is a white salt at ambient temperature that can be added directly to reaction media to carry out the methylation of reactive, acidic analytes^32,33^. The main drawback associated with the employment of TMO is the generation of the tetrafluoroboric acid (HBF_4_) which may interfere with the analysis of acid-sensitive analytes and may cause column degradation over time. However, the effects of this acid can be countered by neutralization with inorganic bases like sodium or potassium bicarbonate during the sample preparation step prior to instrument injection. This approach has proven crucially useful in the analysis of phosphonic and sulfonic acids related to the organophosphorus-based nerve agents^34–37^. In this work we describe our studies of employing TMO as a derivatizing agent in the methylation of a panel of environmentally relevant CPs: 2-chlorophenol (2-CP or 2-chloroanisole), 2,4-dichlorophenol (2,4-DCP or 2,4-dichloroanisole), 2,4,6-trichlorophenol (2,4,6-TCP or 2,4,6-trichloroanisole), pentachlorophenol (PCP or pentachloroanisole) and triclosan (TCS) (Fig. 1b,c). Furthermore, we establish its usefulness in derivatizing these species simultaneously when present at low concentrations (10 μg g^−1^) in six different soil matrices that include Nebraska EPA standard soil, JT Baker^®^ (Baker) and Ottawa sands, Virginia Type A soil, Georgia EPA standard soil and silt.

## Materials and methods

### Chemicals and reagents

All reagents were of analytical grade, were purchased from commercial suppliers, and were used as received. Anhydrous dichloromethane (DCM), anhydrous sodium sulfate and sodium bicarbonate were purchased from VWR (Atlanta, GA.). 2-chlorophenol (2-CP; CAS: *95-57-8*), 2,4-dichlorophenol (2,4-DCP; CAS: *120-83-2*), 2,4,6-trichlorophenol (2,4,6-TCP; CAS: *88-06-2*), pentachlorophenol (PCP; CAS: *87-86-5*) and triclosan (TCS; CAS: *3380-34-5*) were purchased from Aldrich (St. Louis, MO). Trimethyloxonium tetrafluoroborate (TMO^.^BF_4_, > 95%, MW = 147.9, CAS: *420-37-1*) was purchased from TCI America (Portland, OR.). Acrodisc PTFE syringe filters (0.45 μm) were purchased from Pall laboratories (Port Washington, NY). The soils employed for this study were the following: Georgia EPA standard S1-7G-1, Nebraska EPA standard S4.105.9, Baker and Ottawa sands, Silt and Virginia Type A soil, and all belong to the Forensic Science Center (FSC) soil collection at the Lawrence Livermore National Laboratory.

### Soil preparation

Stock solutions for each CP (1 μg^.^mL^−1^) in DCM were prepared and used in the preparation of all soil samples. The solutions were stored at ~ 4 °C in an amber glass scintillation vials and taken out only when preparing the soils. The stock solutions were prepared by making a 1000 μg^.^mL^−1^ solution in DCM of 2-CP, 2,4-DCP, 2,4,6-TCP, PCP and Triclosan, taking 30 μL of this solution and diluting it up to 30 mL with DCM. Afterwards, 300 mg of the soil (Georgia soil (EPA standard S1-7G-1), Nebraska soil (EPA standard S4.105.9), Baker sand, Ottawa sand, Silt and Virginia Type A soil) was treated with 1.5 mL of the stock solution (1 μg^.^mL^−1^) in a 5-mL thick-wall conical reaction vial, vortexed and the DCM was carefully removed in vacuo at ambient temperature with slow rotation in a rotary evaporator to provide a sample matrix containing all five chlorinated phenols at ~ 10 μg^.^g^−1^ concentration each in the soil matrices.^25,36^

### Direct, simultaneous methylation of chlorinated phenols in six separate soils at 10 μg^.^g^−1^

Each soil (300 mg) was made into a suspension in DCM (1 mL) in a glass vial equipped with a stir bar. To the suspension, TMO (10 mg) was added and the resulting mixture was stirred at ambient temperature for 1 h. After which time, the stirring was stopped and the solids allowed to settle to the bottom of the vial. The supernatant DCM layer was passed through a filter disk and transferred to another vial where it was neutralized with saturated NaHCO_3_ (2 mL). The bottom organic layer was then dried over anhydrous Na_2_SO_4_ (10 mg) and concentrated to ~ 50 μL carefully using a nitrogen stream. The volume was then transferred to an autosampler vial with a glass insert and analyzed by Electron Ionization GC–MS (EI-GC–MS).

### Methylation of chlorinated phenols (at 10 μg^.^g^−1^) in soil extracts

Each soil sample (300 mg) was extracted with diethyl ether (2 × 1 mL), the organic extracts were combined and evaporated using a nitrogen stream to yield a residue. All residues from all six different soils were taken up in DCM (1 mL) and treated with TMO (10 mg). The resulting mixture was stirred at ambient temperature for 1 h. After which time, the stirring was stopped and the solids allowed to settle to the bottom of the vial. The supernatant DCM layer was transferred to another vial and neutralized with saturated NaHCO_3_ (2 mL). The bottom organic layer was then dried over anhydrous Na_2_SO_4_, passed through a filter disk and evaporated off using a nitrogen stream. The residue was taken up in DCM (100 μL), placed in an autosampler vial with a glass insert and analyzed by EI-GC–MS.

### Synthesis of methylated standards using TMO

All five methylated products were separately synthesized and purified for use in the MDL determinations in all six soils studied. The general procedure involved dissolving the chlorophenol (1 mmol) in DCM (10 mL) in a 25-mL round bottom flask equipped with a stir bar. The solution was cooled to ~ 4 °C using an ice bath and treated in one portion with TMO (162 mg, 1.1 mmol). After the ice bath is removed, the reaction is stirred vigorously at ambient temperature for 3 h, after which time it was transferred to a separatory funnel and partitioned (DCM//NaHCO_3_), the organic phase was extracted with brine (NaCl/H_2_O, 1 × 40 mL), dried over anhydrous Na_2_SO_4_ and evaporated *in vacuo* to yield in all cases a light brown residue with the exception of 2,4-DCP-Me which was a colorless oil. The residue containing the methylated CP was purified by flash column chromatography using a Biotage Purification System (hexanes → 1:1 hexanes/EtOAc) to give each methylated CP as a solid. 2-CP-Me (124 mg, 87%); 2,4-DCP-Me (140 mg, 79%); 2,4,6-TCP-Me (133 mg, 63%); PCP-Me (185 mg, 66%) and Triclosan-Me (246 mg, 81%).

### GC–MS analysis

A 6890 Agilent GC with a 5975 MS detector equipped with a split/splitless injector was used for the analysis in the splitless mode. The GC column used for the analysis was an Agilent DB-5MS capillary column (30 m × 0.25 mm id × 0.25 μm film thickness). Ultra-high purity helium was used as the carrier gas at 0.8 mL/ min. The injector temperature was 250 °C, and the injection volume was 1 μL. The oven temperature program was as follows: 40 °C, held for 3 min, increased at 8 °C/min to 300 °C, held for 3 min. The MS ion source and quadrupole temperatures were 230 and 150 °C, respectively. Electron ionization was used with ionization energy of 70 eV. The MS was operated to scan from *m/z* 29 to *m/z* 600 in 0.4 s. with a solvent delay of 3.5 min. as described previously.^25,26^

### NMR analysis

Spectra were obtained using a Bruker Avance III 600 MHz instrument equipped with a Bruker TCI 5 mm cryoprobe (Bruker Biospin, Billerica, MA) at 30.0 ± 0.1 °C. ^1^H NMR (600 MHz) and ^13^C NMR (150 MHz) were recorded in CDCl_3_. ^1^H NMR chemical shifts are calibrated with respect to the residual CHCl_3_ singlet centered at 7.26 ppm while for ^13^C NMR the triplet centered at 77.16 ppm from CDCl_3_ was used for the spectral calibration. 2-CP-Me: ^1^H NMR (600 MHz, CDCl_3_) δ 7.36 (dd, *J* = 7.9, 1.7 Hz, 1H), 7.22 (dd, *J* = 9.1, 7.5 Hz, 1H), 6.92 (dd, *J* = 8.3, 1,3 Hz, 1H), 6.89 (td, *J* = 7.6, 1.4 Hz, 1H), 3.90 (s, 3H); ^13^C NMR (151 MHz) δ 155.2, 130.4, 127.9, 122.6, 121.5, 112.3, 56.2; 2,4-DCP-Me: ^1^H NMR (600 MHz, CDCl_3_) δ 7.36 (d, *J* = 2.5 Hz, 1H), 7.19 (dd, *J* = 8.8, 2.5 Hz, 1H), 6.84 (d, *J* = 8.8 Hz, 1H), 3.88 (s, 3H); ^13^C NMR (151 MHz) δ 154.1, 130.1, 127.7, 125.8, 123.5, 112.9, 56.5; 2,4,6-TCP-Me: ^1^H NMR (600 MHz, CDCl_3_) δ 7.31 (s, 2H), 3.89 (s, 3H); ^13^C NMR (151 MHz) δ 151.6, 130.2, 129.7, 129.0, 60.0; PCP-Me: ^1^H NMR (600 MHz, CDCl_3_) δ 3.92 (s, 3H); ^13^C NMR (151 MHz) δ 152.8, 132.0, 129.6, 128.5, 61.1; Triclosan-Me: ^1^H NMR (600 MHz, CDCl_3_) δ 7.43 (d, *J* = 2.5 Hz, 1H), 7.10 (dd, *J* = 8.8, 2.5 Hz, 1H), 6.98 (d, *J* = 2.3 Hz, 1H), 6.91 (dd, *J* = 8.5, 2.3 Hz, 1H), 6.84 (d, *J* = 8.5 Hz, 1H), 6.68 (d, *J* = 8.8 Hz, 1H), 3.82 (s, 3H); ^13^C NMR (151 MHz) δ 152.2, 151.6, 143.3, 130.5, 130.4, 128.4, 128.0, 125.0, 121.3, 121.1, 118.7, 113.8, 56.4. All ^1^H and ^13^C NMR spectra are provided in the Supporting Information (Pages S2-S6).

## Results and discussion

When devising the experimental setup for the methylation of the chlorinated phenols, we concentrated on the following five members of this class of compounds mainly as a result of their wide environmental prevalence and industrial use^3,6^: 2-chlorophenol (2-CP), 2,4-dichlorophenol (2,4-DCP), 2,4,6-trichlorophenol (2,4,6-TCP), pentachlorophenol (PCP) and Triclosan (TCS, CH-3565 or Lexol 300) (Fig. 1). Analysis of CPs often involves the extraction of these species out the matrix by various methods like Soxhlet^38^ and microwave-assisted extraction^39^. Alternatively, one of the most common extraction techniqes for CPs from soils has become solid–liquid extraction (SLE) in which a polar, aprotic solvent like DCM, diethyl ether and even n-hexanes is employed for isolating these species^40,41^. Once their extraction is completed, then their direct detection or detection after derivatization follows. One such method is the use of diazomethane (DM) whereby the CPs are methylated to yield products that can be unambiguously corroborated using the GC’s mass spectral library^42,43^. We decided to examine two specific methods to carry out the methylation of the CPs for our studies, the first one involves the direct treatment of the spiked soil with TMO in DCM, while the second one involves an initial extraction step of the CPs out of the soils followed by treatment with TMO.

**Figure 2:**
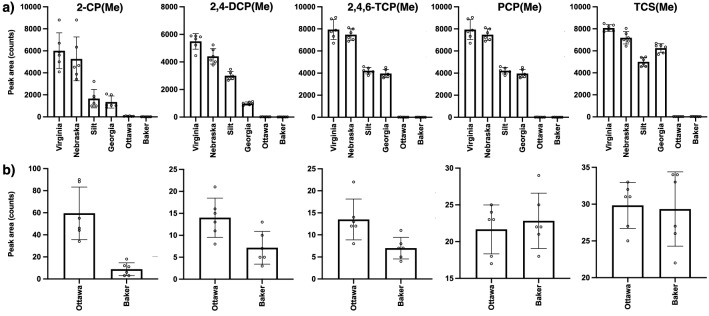
**(a)** Average (n = 6) peak areas (± the standard deviation) for all five methylated CPs in all six soils studied in this work when directly derivatized with TMO. **(b)** Expansion of graph areas for the derivatization on the Ottawa and Baker sand matrices for all CPs.

Our first derivatization protocol involved the direct treatment of the soil as a suspension in DCM with TMO. Previous work reported that the insolubility of the TMO salt in DCM does not represent a problem in the process and methylation in this case of phosphonic acids still occurred in the suspension^34^. The direct derivatization of each soil yielded interesting results that clearly reflected the vast differences in composition for each matrix and how it affected the methylation of individual CPs. Generally, the higher molecular weight CPs (i.e. 2,4,6-TCP, PCP and TCS) tended to provide higher magnitude signals than those of 2-CP and 2,4-DCP which are our two lowest molecular weights in our panel (Fig. 2). Additionally, regarding the derivatization’s effectiveness in the soils, one can observe that the method performed relatively well in all soils with the exception of the sand matrices (Ottawa and JT Baker sand) despite their lowest total organic content. We conjecture that this could be a result of two factors or a combination of both. The first factor been a sequestering ability of the sand matrix in the interior of the porous material effectively shielding the CP from the reagent. The other could be the reactivity of the sand itself at its surface (silanols) with TMO effectively depleting it from the mixture. Although derivatization in the remaining soils appeared to have gone pretty well, it can be observed that the method performed slightly better in the Virginia and Nebraska soils for the lower molecular weight CPs (2-CP and 2,4-DCP) than in the Georgia soil or Silt matrix. This can be attributed to more difficult physical disposition of the Georgia and Silt matrices that aside from containing high clay concentration are very fine powders^36^. Interestingly, derivatization of the other three CPs across the Virginia, Nebraska, Georgia and Silt soil samples is comparable and this could be attributed to the much higher molecular weight of these and response originating from their additional Cl atoms.

**Figure 3:**
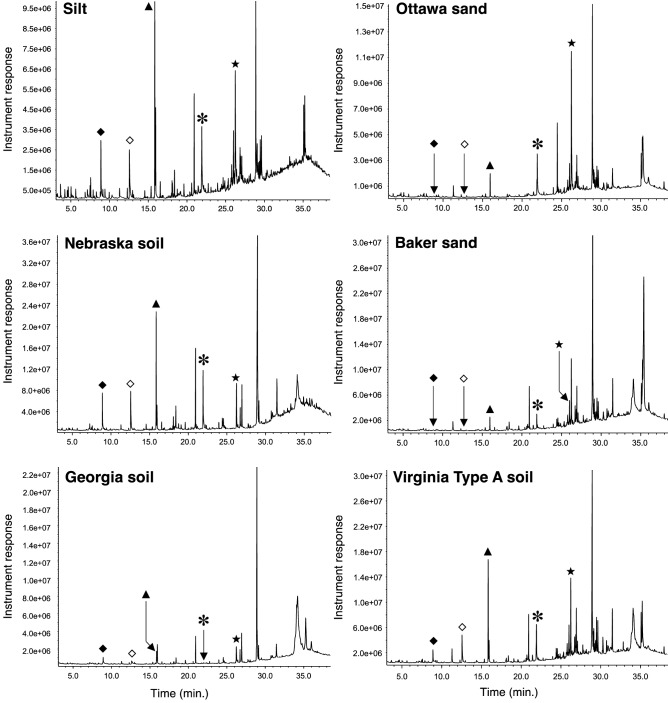
GC–MS chromatograms showing the extraction of all five CPs from all soils (at a 10 μg^.^g^-1^ concentration each) from all soils using diethyl ether followed by filtration. All five CPs, 2-CP (filled diamond), 2,4-DCP (open diamond), 2,4,6-TCP (filled triangle), PCP (asterisk) and TCS (star) were identified using the instrument’s NIST database. Ion extraction was needed to obtain a signal for further processing for 2-CP and 2,4-DCP in both, Ottawa and Baker sand matrices.

**Figure 4:**
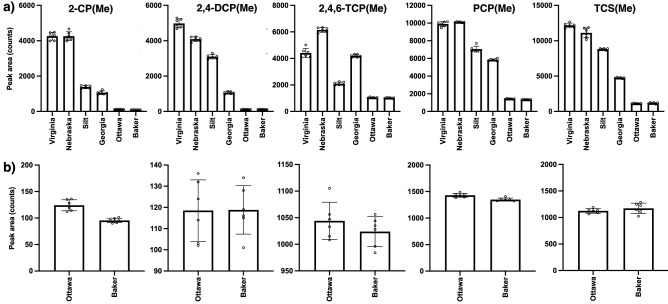
**(a)** Average (n = 6) peak areas (± the standard deviation) for all five methylated CPs in all six soils studied in this work after the extraction (w/diethyl ether) followed by derivatization with TMO protocol was employed; **(b)** expansion of graph areas for the derivatization on the Ottawa and Baker sand matrices for all CPs.

**Figure 5:**
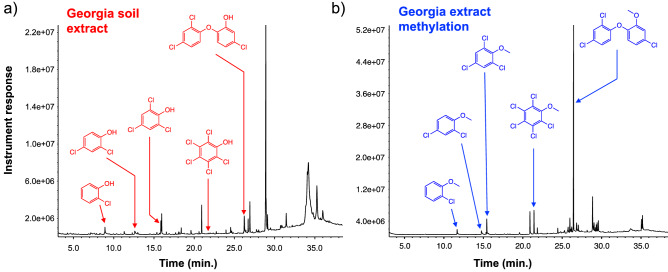
GC chromatograms showing the protocol’s modification involving extraction using diethyl ether **(a)** followed by the derivatization to yield the methylated CPs **(b)** in Georgia EPA standard soil.

In an effort to improve the derivatization efficiency of the CPs, particularly within the context of the sand matrices, we explored a version of the protocol that involves an initial organic extraction of the CPs followed by the derivatization. As a first step, we employed DCM as the extracting medium but found that upon careful evaporation of the organic to yield a residue containing the CPs, loss of substantial quantity of the analytes plagued this approach. Therefore, we decided to choose another polar, aprotic organic solvent that could still serve as a good extractant for the CPs and possess a lower boiling point than DCM, so that evaporation of such will be rapid and would not compromise the concentration of the extracted CPs. To this end, diethyl ether proved to be the ideal organic solvent for this purpose as shown in Fig. 3 for all six soils employed and also for its previous uses during SLE extractions of CPs from various matrices^44,45^. Evaporation of the ether followed by treatment of the residue with TMO resulted in the methylation of the CPs (Fig. 4). Interestingly, extraction was found to improve the overall derivatization of the CPs relative to the direct methylation protocol, a modification that has found success in other analyte derivatizations such as acylations^46^. In fact, prior extraction of the CPs in the sand samples increased the detection of the methylated adducts, however, still in a much lower concentration than in the other soils (Fig. 4). Among the two types of sand samples, we expected some difference as one type of sand particles are smoother (Ottawa sand) as a result of their industrial production while particles from Baker sand are not only smaller in size but feature highly irregular, coarse shapes. Nevertheless, both types of sands can still trap analytes in their interior and as such can already create problems in their overall derivatizations. In addition to this phenomenon, the surface of the sand particles may also be reactive towards a strong electrophile such as TMO, thus depleting the agent availability for the derivatization^6^. An example of the effectiveness of this modification of the protocol can be appreciated in Fig. 5, where the extraction of all five CPs from the Georgia soil matrix (Fig. 5a), was followed by methylation to yield a very clear GC chromatogram of the products (Fig. 5b). The same observation was found to be true in the remaining soils (See Supporting Information, Pages S7–S8).

The MDL values for the methylation protocol was determined for each of the five CPs accross all six examined soils and these are presented in Table 1. A general assessment of the values in Table 1 indicate that lower limits of detection for the method are clearly observed for soil matrices with low organic/clay content such as Ottawa and JT Baker sand and to some extent the Georgia EPA standard soil. In this specific first block of analysis, it is interesting to find that the MDL values found for the Georgia EPA standard soil are comparable to those found for the sand matrices despite possessing a larger total organic content (TOC)^29^. One reason for the lower value for the Georgia EPA standard soil could be on its finer particle size relative to the Virginia Type A and the Nebraska EPA standard soils that consist of larger, more granular particles that could be sites for analyte sequestration, in addition to the larger silt contents exhibited by these latter soils in their composition. As already discussed, larger MDL values are observed for the Virginia Type A soil, Nebraska EPA standard soil and silt matrices and this can be attributed to aside from the soils’ larger granularity in the first two cases and the ultra-fine nature of the particles in silt. In addition to the particle granularity exhibited by the Virginia Type A and the Nebraska EPA standard soils, these soils contain the most silt percentages, 28% and 58% respectively^29^, of all the matrices used in this work aside from silt itself.

**Table 1:** Calculated MDL values for each methylated CP in each soil matrix.

Soil matrix CP (ng^.^mL^−1^)	Nebraska (EPA S4.105.9)	Virginia type A	Silt	Georgia EPA S1-7G-1	Ottawa sand	JT Baker sand
2-CP	8.2	15.6	16.0	9.0	5.9	4.1
2,4-DCP	12.7	9.6	21.8	5.1	6.4	7.0
2,4,6-TCP	13.4	12.2	15.0	8.5	6.7	5.3
PCP	8.2	11.7	15.4	8.2	8.5	5.1
TCS	15.0	12.1	12.2	13.2	4.5	5.7

## Conclusions

A methylation method for the chemical derivatization of five environmentally-relevant CPs in six different soils has been developed using the methylating salt trimethyloxonium tetrafluoroborate (TMO). Two different modes for carrying out the method were studied. The first one involved the direct treatment of each soil suspension in DCM with TMO while the second one featured a preliminary soil extraction step using diethyl ether to isolate the CPs, followed by derivatization with TMO. Two general observations about the protocol’s performance can be discerned, one is that the lower molecular weight CPs (i.e*.* 2-chlorophenol and 2,4-dichlorophenol) consistently provided lower signal response in the GC analysis relative to 2,4,6-trichlorophenol, pentachlorophenol and triclosan. The second observation is that the direct methylation method showed low performance in both sand matrices studied (Ottawa and Baker sands) relative to the other four soils (*i.e.* Nebraska EPA standard soil, Virginia type A soil, Georgia EPA standard soil and Silt). In contrast, the protocol was found to perform better in modifying the CPs when these were initially extracted and derivatizated with TMO. The MDL values for each methylated CP were determined and lower values were found in the range of 4.1–13.2 ng^.^mL^–1^ for both sand matrices as well as for the Georgia EPA standard soil, while larger values in the range of 8.2–21.8 ng^.^mL^−1^ were found for the Virginia Type soil, Nebraska EPA standard soil and silt.

## Supplementary Information

Supplementary Information.
