# Supplementary material for: Trimethyloxonium-mediated methylation strategies for the rapid and simultaneous analysis of chlorinated phenols in various soils by electron impact gas chromatography–mass spectrometry
Source: Sci Rep. 2022 Jan 26;12:1401. doi: 10.1038/s41598-022-05463-w (PMC8792036; doi:10.1038/s41598-022-05463-w)
Supplement: Supplementary file 1 — Supplementary Information. [file 41598_2022_5463_MOESM1_ESM.pdf]

-SUPPORTING INFORMATION-

Trimethyloxonium-mediated methylation strategies for the analysis of chlorinated phenols in soils by Electron Impact Gas Chromatography-Mass Spectrometry

Carlos A. Valdez*, Edmund P. Salazar and Roald N. Leif
Lawrence Livermore National Laboratory

-Table of Contents-

Content	Page
NMR acquisition details	S2
Figure S1. ^{1}H NMR of O-methylated 2-CP (2-chloroanisole)	S2
Figure S2. ^{13}C NMR of O-methylated 2-CP (2-chloroanisole)	S2
Figure S3. ^{1}H NMR of O-methylated 2,4-DCP (2,4-dichloroanisole)	S3
Figure S4. ^{13}C NMR of O-methylated 2,4-DCP (2,4-dichloroanisole)	S3
Figure S5. ^{1}H NMR of O-methylated 2,4,6-TCP (2,4,6-trichloroanisole)	S4
Figure S6. ^{13}C NMR of O-methylated 2,4,6-TCP (2,4,6-trichloroanisole)	S4
Figure S7. ^{1}H NMR of O-methylated PCP (pentachloroanisole)	S5
Figure S8. ^{13}C NMR of O-methylated PCP (pentachloroanisole)	S5
Figure S9. ^{1}H NMR of O-methylated TCS	S6
Figure S10. ^{13}C NMR of O-methylated TCS	S6
Figure S11. Methylation of CPs in Virginia soil (GC-MS)	S7
Figure S12. Methylation of CPs in Ottawa sand	S7
Figure S13. Methylation of CPs in Nebraska soil	S7
Figure S14. Methylation of CPs in Baker sand	S8
Figure S15. Methylation of CPs in Silt	S8
MDL calculations details	S9-S12

NMR acquisition details

Spectra were obtained using a Bruker Avance III 600 MHz instrument equipped with a Bruker TCI 5 mm cryoprobe (Bruker Biospin, Billerica, MA) at 30.0 ± 0.1 °C. ^{1}H NMR (600 MHz) and ^{13}C NMR (150 MHz) were recorded in $CDCl_3$. ^{1}H NMR chemical shifts are calibrated with respect to the residual $CHCl_3$ singlet centered at 7.26 ppm while for ^{13}C NMR the triplet centered at 77.16 ppm from $CDCl_3$ was used for the spectral calibration.

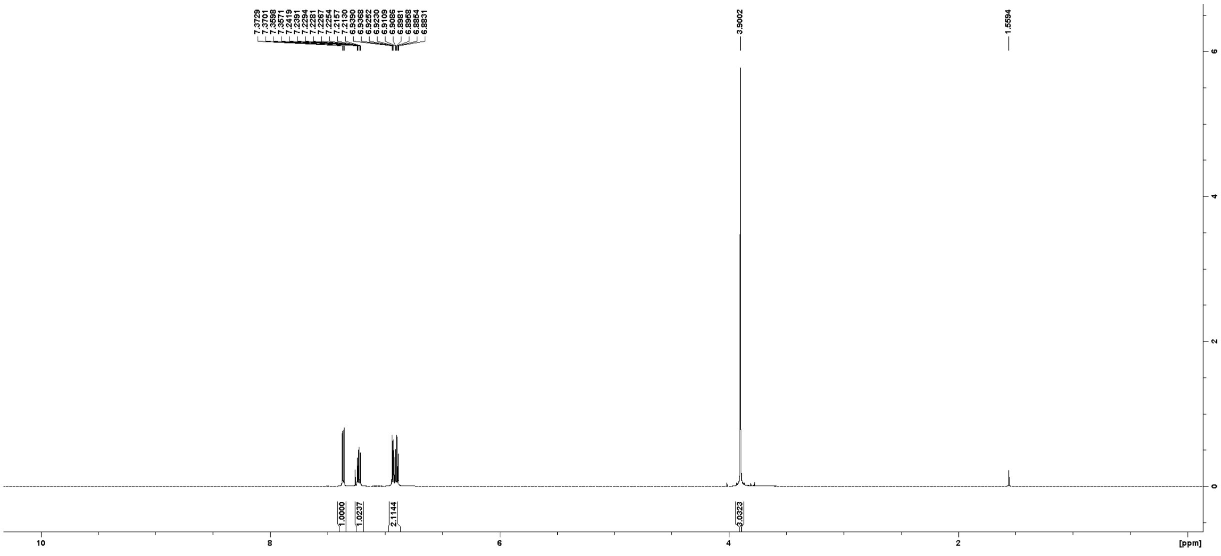

Figure S1. ^{1}H NMR of O-methylated 2-CP (2-chloroanisole).

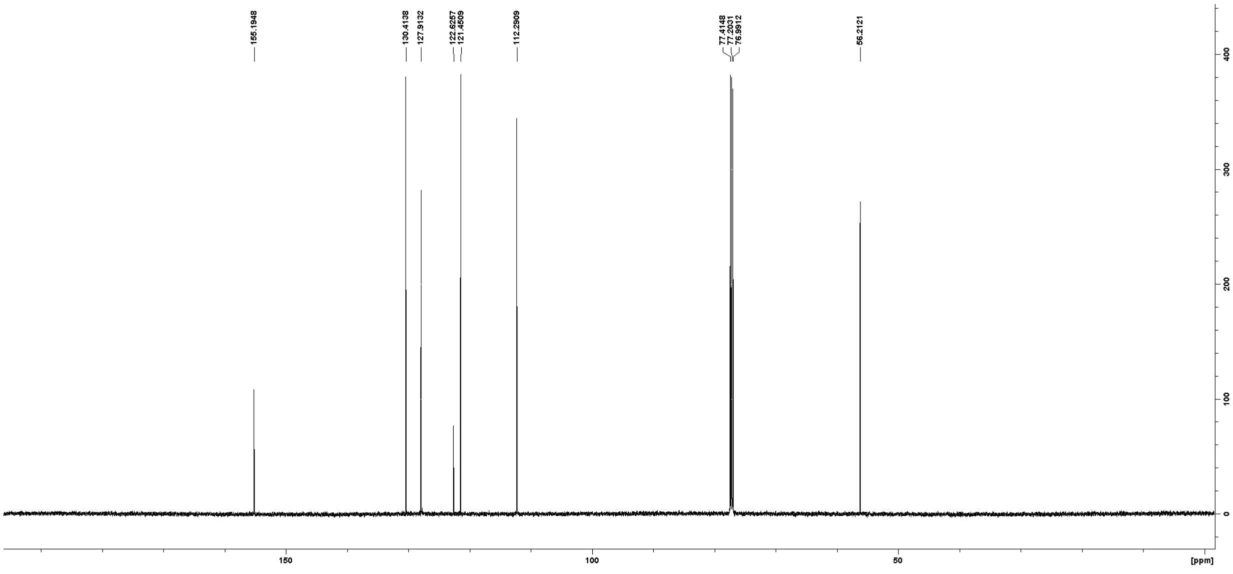

Figure S2. ^{13}C NMR of O-methylated 2-CP (2-chloroanisole).

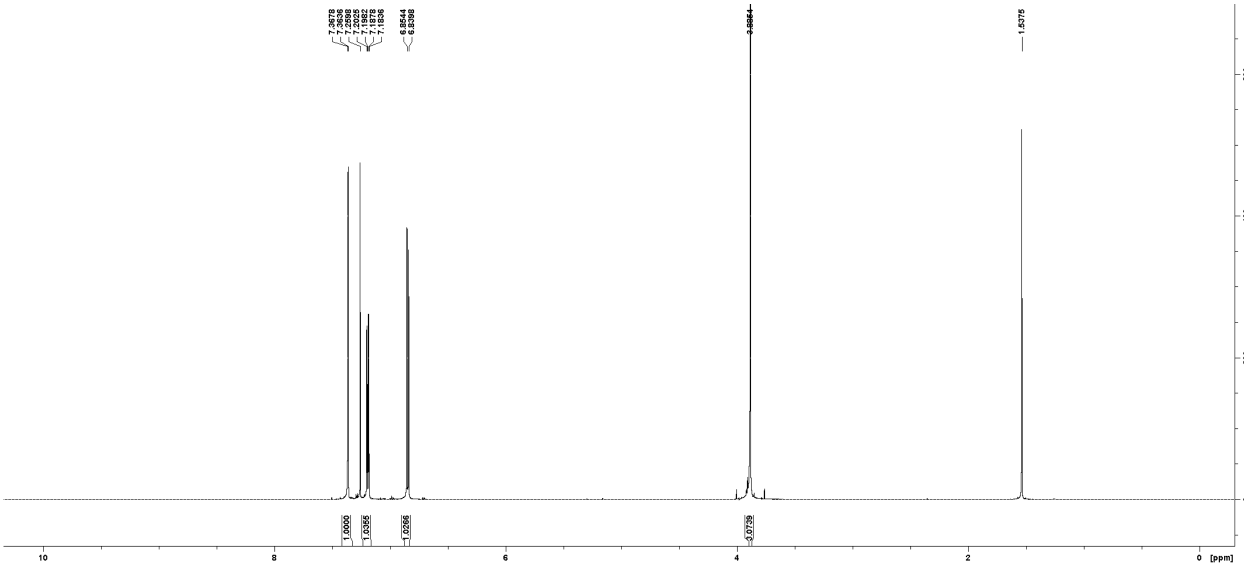

Figure S3. ^{1}H NMR of O-methylated 2,4-DCP (2,4-dichloroanisole).

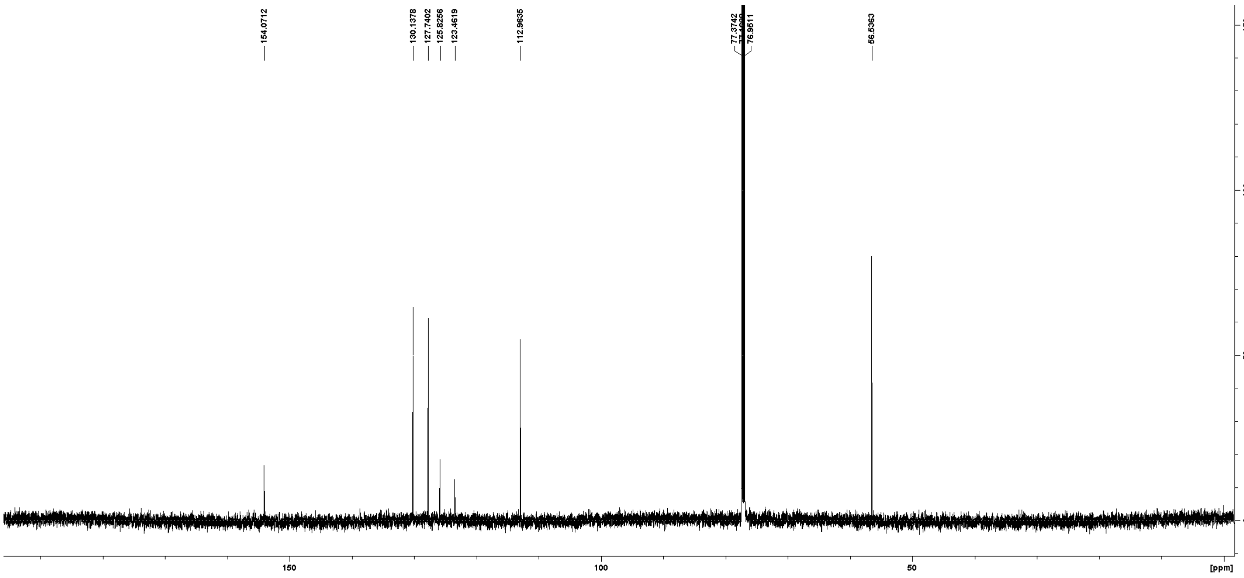

Figure S4. ^{13}C NMR of O-methylated 2,4-DCP (2,4-dichloroanisole).

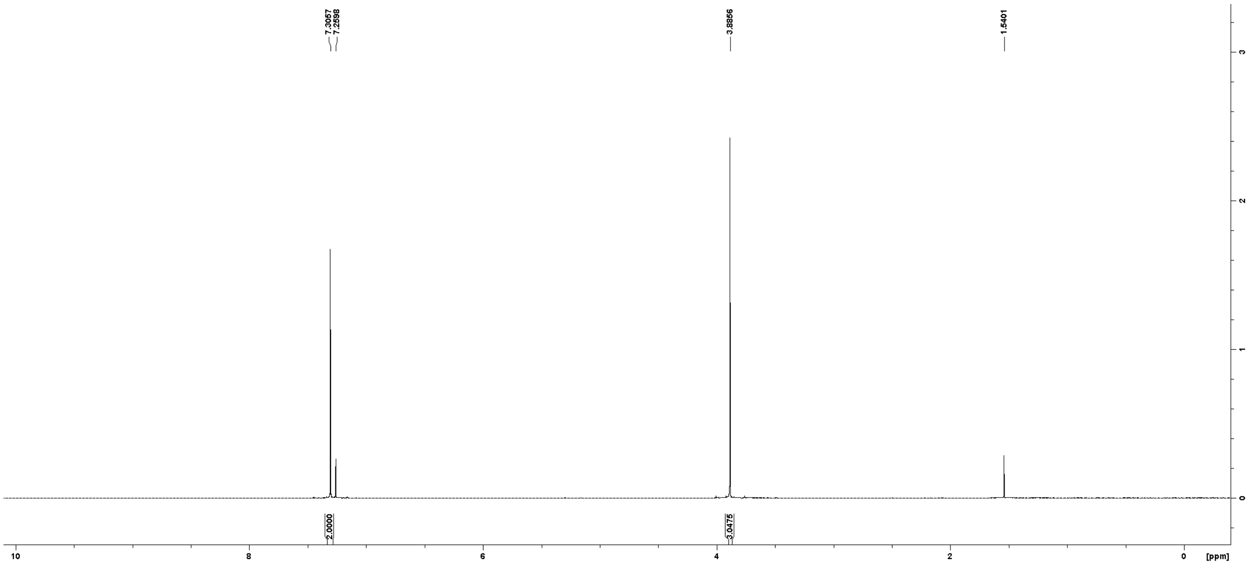

Figure S5. ^{1}H NMR of O-methylated 2,4,6-TCP (2,4,6-trichloroanisole).

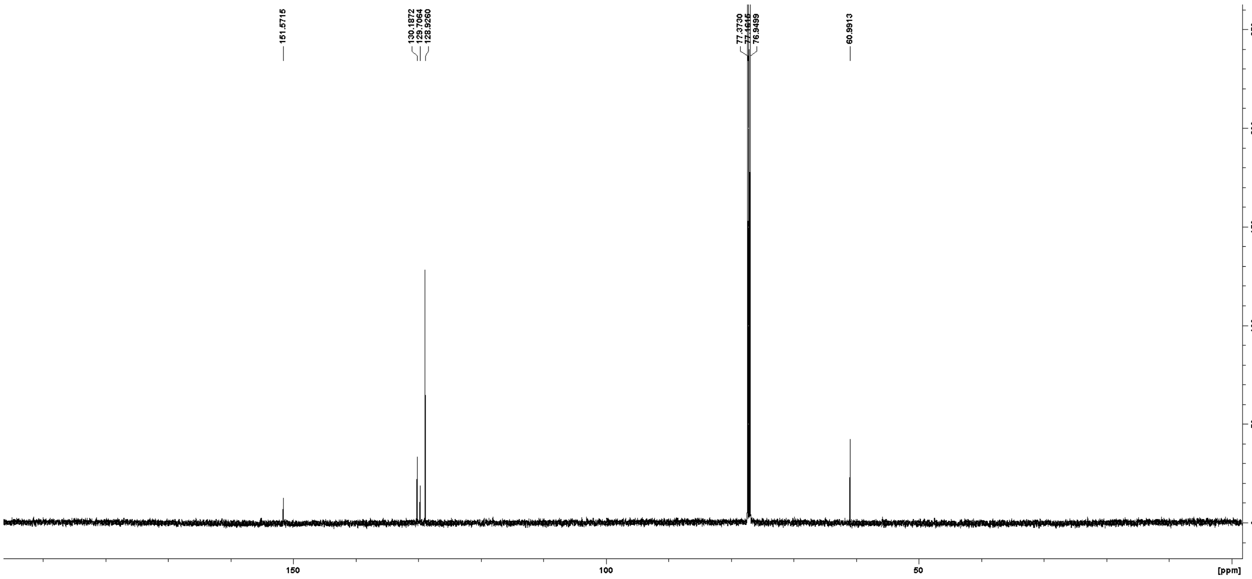

Figure S6. ^{13}C NMR of O-methylated 2,4,6-TCP (2,4,6-trichloroanisole).

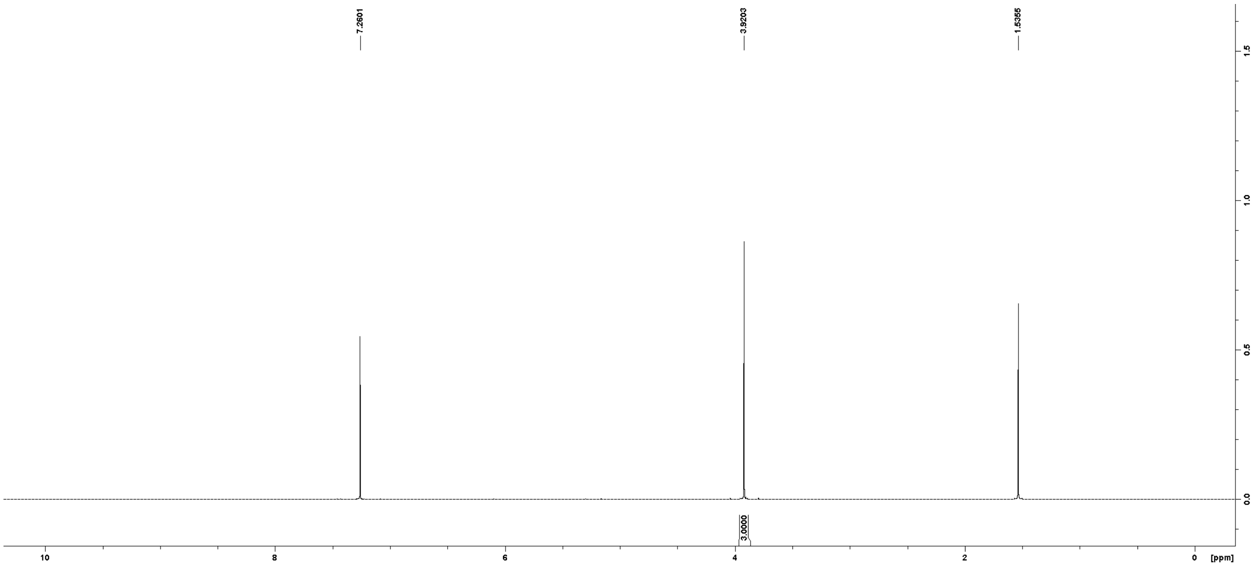

Figure S7. ^{1}H NMR of O-methylated PCP (pentachloroanisole).

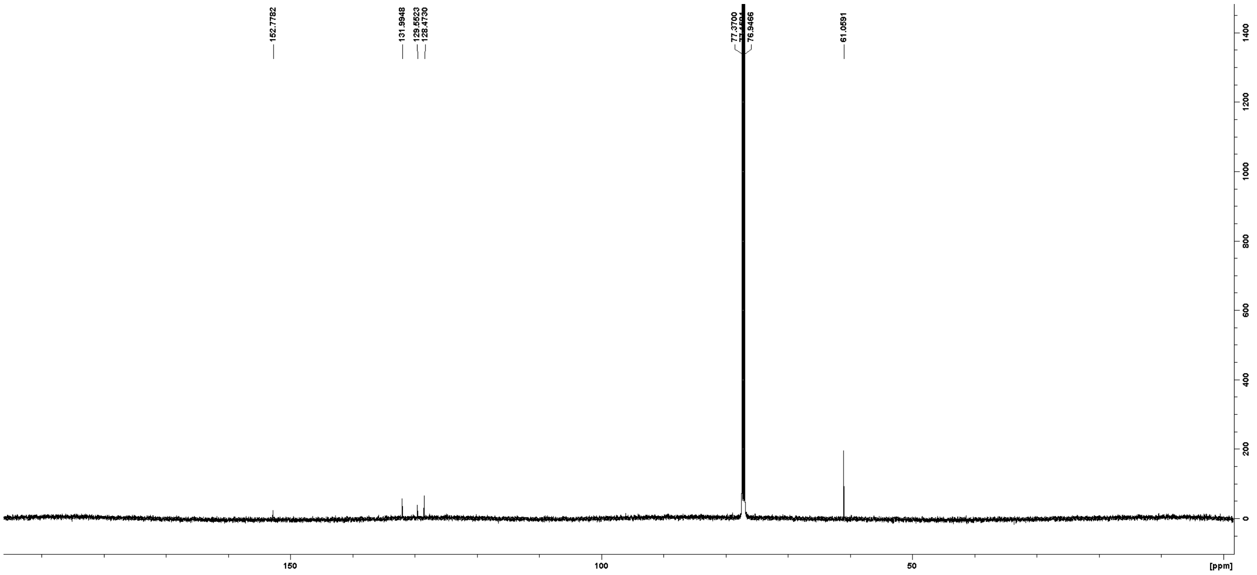

Figure S8. ^{13}C NMR of O-methylated PCP (pentachloroanisole).

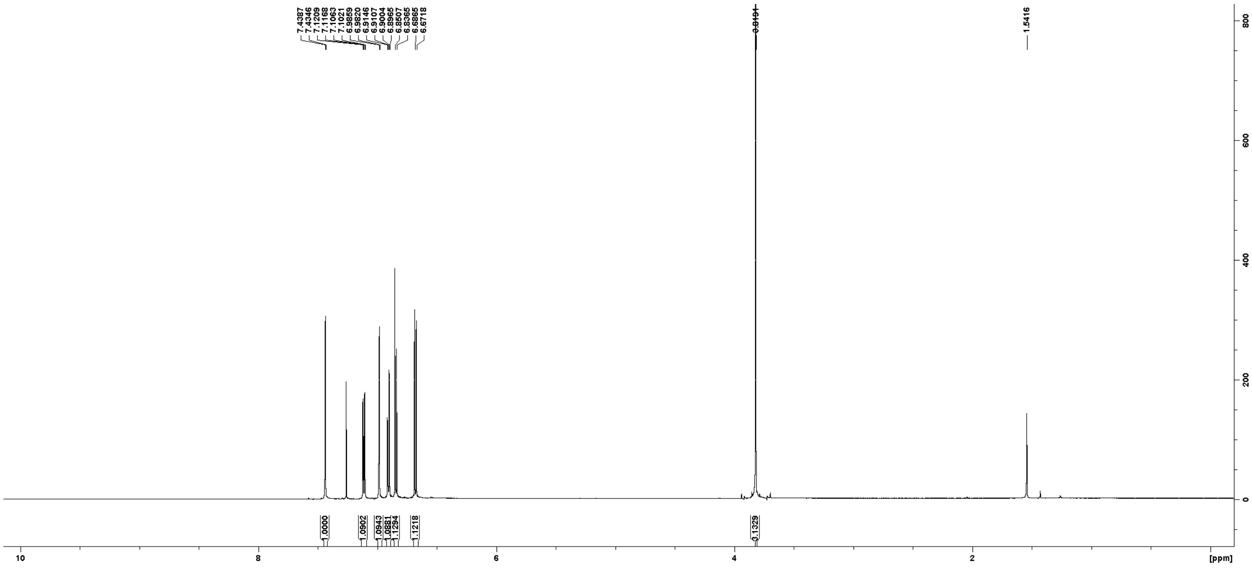

Figure S9. ^{1}H NMR of O-methylated TCS.

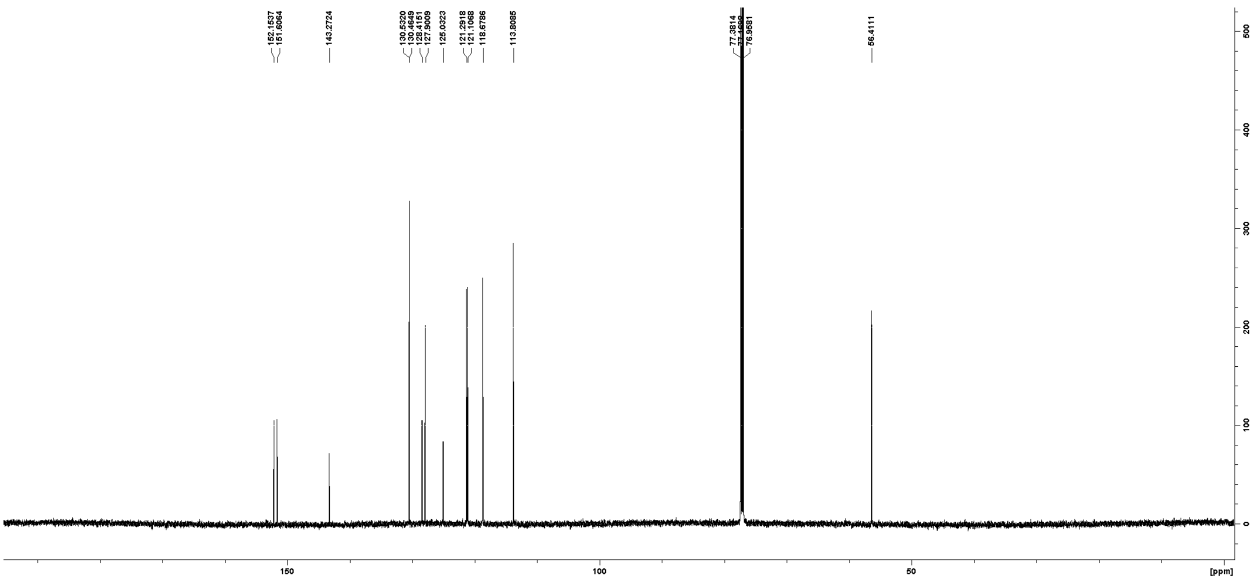

Figure S10. ^{13}C NMR of O-methylated TCS.

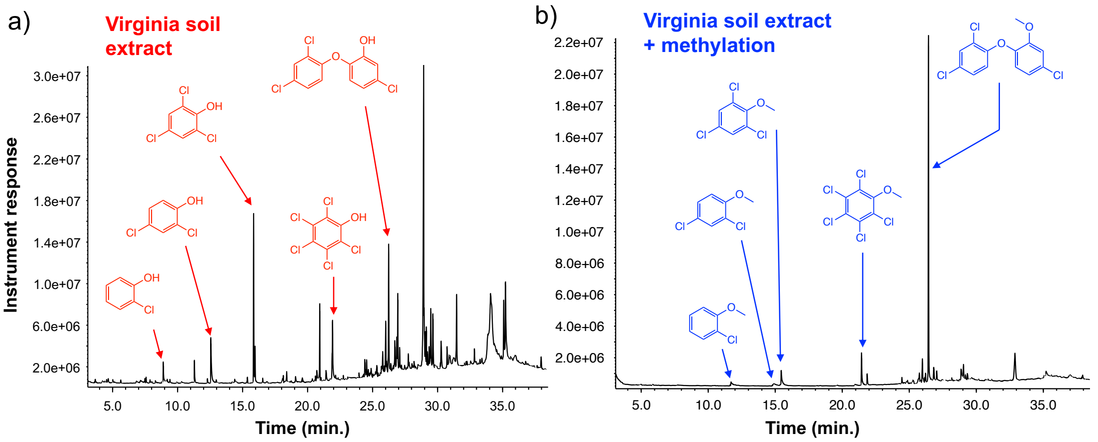

Figure S11. GC chromatograms showing the protocol's modification involving extraction from Virginia Type A soil with diethyl ether (a) followed by the derivatization (b) to yield the methylated CPs.

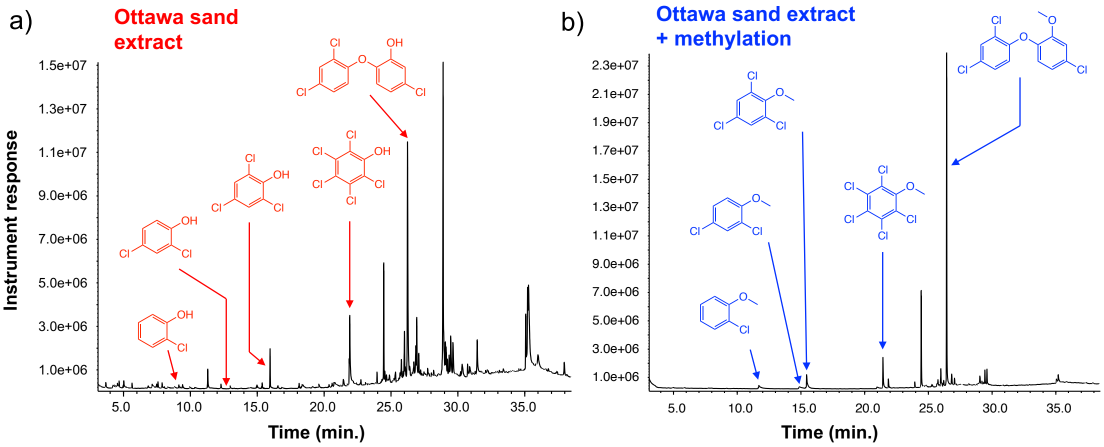

Figure S12. GC chromatograms showing the protocol's modification involving extraction from Ottawa sand with diethyl ether (a) followed by the derivatization (b) to yield the methylated CPs.

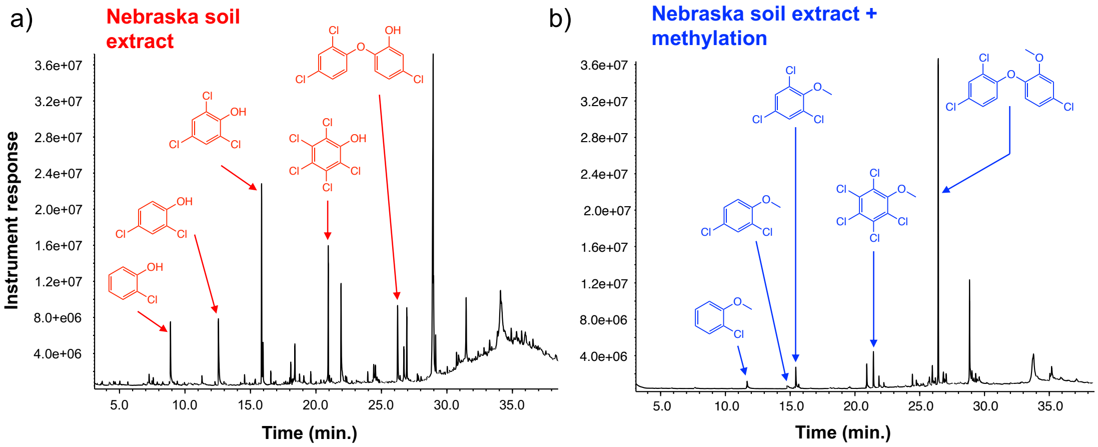

Figure S13. GC chromatograms showing the protocol's modification involving extraction from Nebraska EPA standard soil with diethyl ether (a) followed by the derivatization (b) to yield the methylated CPs.

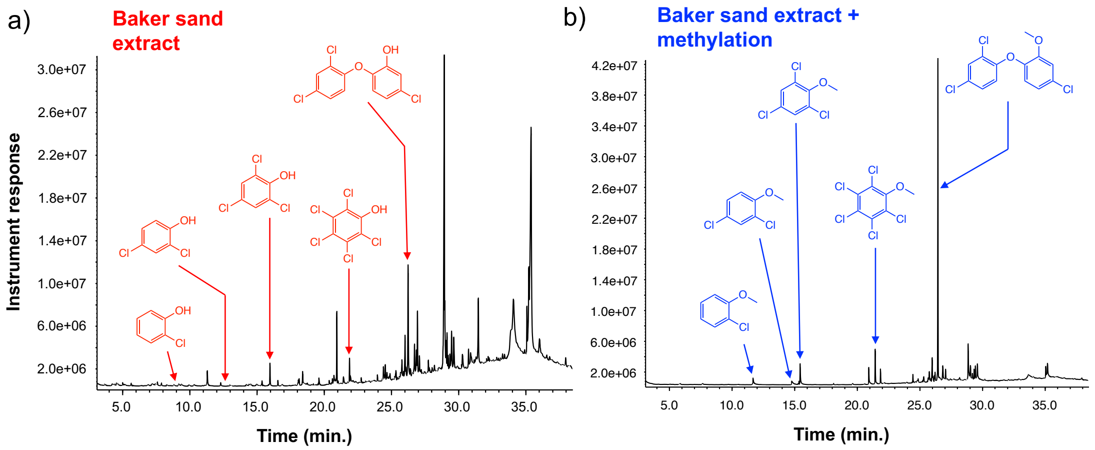

Figure S14. GC chromatograms showing the protocol's modification involving extraction from Baker sand with diethyl ether (a) followed by the derivatization (b) to yield the methylated CPs.

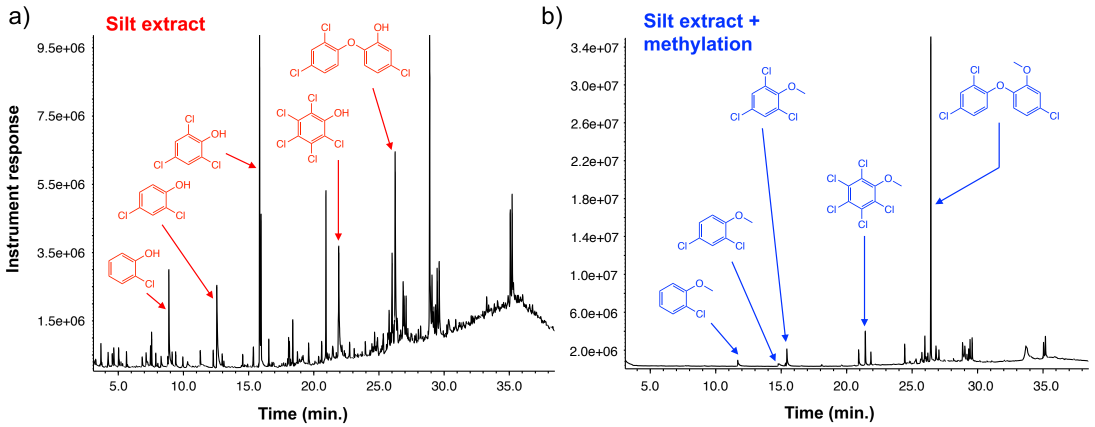

Figure S15. GC chromatograms showing the protocol's modification involving extraction from silt with diethyl ether (a) followed by the derivatization (b) to yield the methylated CPs.

MDL calculations for methylated CPs in all soils

Individual soil matrices were prepared by adding dichloromethane (1 mL) to 300 mg of the specific soil, followed by vortexing the mixtures for 1 minute. These soil extracts were used to determine the sample matrix MDL values for each methylated CP. The MDL for each CP in each specific sample matrix for the three soil types was determined according to the Environmental Protection Agency (EPA) document (2016 EPA document *Definition and Procedure for the Determination of the Method Detection Limit, Revision 2* (EPA 821-R-16-006)) and is as follows:

1. Analyze the sample matrix to determine the native (background) concentration of the analyte(s) of interest;
2. If the response for the native concentration is at a signal-to-noise ratio of approximately 5-20, determine the matrix-specific MDL according to Section 2 (EPA 821-R-16-006) but without spiking additional analyte;
3. Calculate MDL_b using the method blanks, not the sample matrix.
4. If the signal-to-noise ratio is less than 5, then the analyte(s) should be spiked into the sample matrix to obtain a concentration that will give results with a signal-to-noise ratio of approximately 10-20.
5. If the analytes(s) of interest have signal-to-noise ratio(s) greater than approximately 20, then the resulting MDL is likely to be biased high.

MDL calibration curve for all CPs in Ottawa Sand

The method detection limit (MDL) for the methylated versions of 2-CP, 2,4-DCP, 2,4,6-TCP, PCP and TCS was determined to be 5.9, 6.4, 6.7, 8.5 and 4.5 ng/mL (7 replicates using the single-tailed 99th percentile *t* statistic) respectively. The MDL was determined following the guidelines in EPA document EPA 821-R-16-006 "Definition and Procedure for the Determination of the Method Detection Limit, Revision 2," December, 2016. A linear 8-point calibration curve was generated for this study, covering a CP calibration range from 50 to 800 ng/mL.

				**2-CP**	**2,4-DCP**	**2,4,6-TCP**	**PCP**	**TCS**
Date Acquired	Data File Name	Sample Name	Misc Info	ng/mL	ng/mL	ng/mL	ng/mL	ng/mL
6/15/21 15:51	CW004339.D	Ottawa L1	Rep 1	50	48	48	42	51
6/15/21 16:34	CW004340.D	Ottawa L1	Rep 2	46	50	46	46	51
6/15/21 17:18	CW004341.D	Ottawa L1	Rep 3	46	45	49	41	47
6/15/21 18:01	CW004342.D	Ottawa L1	Rep 4	49	50	49	41	50
6/15/21 18:45	CW004343.D	Ottawa L1	Rep 5	49	45	52	38	51
6/15/21 19:28	CW004344.D	Ottawa L1	Rep 6	45	50	47	41	50
6/15/21 20:11	CW004345.D	Ottawa L1	Rep 7	48	48	45	44	49
			StDev	1.9	2.0	2.1	2.7	1.4
		Ottawa L1	**MDL**	5.9	6.4	6.7	8.5	4.5

MDL calibration curve for all CPs in Baker Sand

The method detection limit (MDL) for the methylated versions of 2-CP, 2,4-DCP, 2,4,6-TCP, PCP and TCS was determined to be 4.1, 7.0, 5.3, 5.1 and 5.7 ng/mL (7 replicates using the single-tailed 99th percentile *t* statistic) respectively. The MDL was determined following the guidelines in EPA document EPA 821-R-16-006 "Definition and Procedure for the Determination of the Method Detection Limit, Revision 2," December, 2016. A linear 8-point calibration curve was generated for this study, covering a CP calibration range from 50 to 800 ng/mL.

				**2-CP**	**2,4-DCP**	**2,4,6-TCP**	**PCP**	**TCS**
Date Acquired	Data File Name	Sample Name	Misc Info	ng/mL	ng/mL	ng/mL	ng/mL	ng/mL
6/16/21 1:14	CW004352.D	Sand L1	Rep 1	48	48	48	37	55
6/16/21 1:57	CW004353.D	Sand L1	Rep 2	46	48	49	37	52
6/16/21 2:40	CW004354.D	Sand L1	Rep 3	49	44	47	42	55
6/16/21 3:24	CW004355.D	Sand L1	Rep 4	48	43	45	39	57
6/16/21 4:07	CW004356.D	Sand L1	Rep 5	46	45	45	39	56
6/16/21 4:50	CW004357.D	Sand L1	Rep 6	48	46	47	37	52
6/16/21 5:33	CW004358.D	Sand L1	Rep 7	50	43	45	39	54
			StDev	1.3	2.2	1.7	1.6	1.8
		Sand	**MDL**	4.1	7.0	5.3	5.1	5.7

MDL calibration curve for all CPs in Georgia soil (Std. EPA S1-7G-1)

The method detection limit (MDL) for the methylated versions of 2-CP, 2,4-DCP, 2,4,6-TCP, PCP and TCS was determined to be 9.0, 5.1, 8.5, 8.2 and 13.2 ng/mL (7 replicates using the single-tailed 99th percentile *t* statistic) respectively. The MDL was determined following the guidelines in EPA document EPA 821-R-16-006 "Definition and Procedure for the Determination of the Method Detection Limit, Revision 2," December, 2016. A linear 8-point calibration curve was generated for this study, covering a CP calibration range from 50 to 800 ng/mL.

				**2-CP**	**2,4-DCP**	**2,4,6-TCP**	**PCP**	**TCS**
Date Acquired	Data File Name	Sample Name	Misc Info	ng/mL	ng/mL	ng/mL	ng/mL	ng/mL
6/16/21 11:03	CW004365.D	Georgia L1	Rep 1	55	54	62	57	78
6/16/21 11:47	CW004366.D	Georgia L1	Rep 2	50	55	58	57	67
6/16/21 12:30	CW004367.D	Georgia L1	Rep 3	46	52	58	53	68
6/16/21 13:14	CW004368.D	Georgia L1	Rep 4	48	54	59	52	73
6/16/21 13:57	CW004369.D	Georgia L1	Rep 5	50	55	54	51	67
6/16/21 14:41	CW004370.D	Georgia L1	Rep 6	52	51	54	50	66
6/16/21 15:24	CW004371.D	Georgia L1	Rep 7	49	54	56	53	70
			StDev	2.9	1.6	2.7	2.6	4.2
		Georgia L1	**MDL**	9.0	5.1	8.5	8.2	13.2

MDL calibration curve for all CPs in Nebraska soil (Std. EPA S4.105.9)

The method detection limit (MDL) for the methylated versions of 2-CP, 2,4-DCP, 2,4,6-TCP, PCP and TCS was determined to be 8.2, 12.7, 13.4, 8.2 and 15.0 ng/mL (7 replicates using the single-tailed 99th percentile *t* statistic) respectively. The MDL was determined following the guidelines in EPA document EPA 821-R-16-006 "Definition and Procedure for the Determination of the Method Detection Limit, Revision 2," December, 2016. A linear 8-point calibration curve was generated for this study, covering a CP calibration range from 50 to 800 ng/mL.

				**2-CP**	**2,4-DCP**	**2,4,6-TCP**	**PCP**	**TCS**
Date Acquired	Data File Name	Sample Name	Misc Info	ng/mL	ng/mL	ng/mL	ng/mL	ng/mL
6/16/21 20:30	CW004378.D	Nebraska L1	Rep 1	47	60	60	49	67
6/16/21 21:14	CW004379.D	Nebraska L1	Rep 2	45	60	52	45	68
6/16/21 21:57	CW004380.D	Nebraska L1	Rep 3	43	59	48	50	69
6/16/21 22:41	CW004381.D	Nebraska L1	Rep 4	42	50	50	49	56
6/16/21 23:24	CW004382.D	Nebraska L1	Rep 5	49	62	47	44	59
6/17/21 0:08	CW004383.D	Nebraska L1	Rep 6	43	60	50	45	62
6/17/21 0:51	CW004384.D	Nebraska L1	Rep 7	46	54	52	44	64
			StDev	2.6	4.1	4.3	2.6	4.8
		Nebraska	**MDL**	8.2	12.7	13.4	8.2	15.0

MDL calibration curve for all CPs in Virginia Type A soil

The method detection limit (MDL) for the methylated versions of 2-CP, 2,4-DCP, 2,4,6-TCP, PCP and TCS was determined to be 15.6, 9.6, 12.2, 11.7 and 12.1 ng/mL (7 replicates using the single-tailed 99th percentile *t* statistic) respectively. The MDL was determined following the guidelines in EPA document EPA 821-R-16-006 "Definition and Procedure for the Determination of the Method Detection Limit, Revision 2," December, 2016. A linear 8-point calibration curve was generated for this study, covering a CP calibration range from 50 to 800 ng/mL.

				**2-CP**	**2,4-DCP**	**2,4,6-TCP**	**PCP**	**TCS**
Date Acquired	Data File Name	Sample Name	Misc Info	ng/mL	ng/mL	ng/mL	ng/mL	ng/mL
6/17/21 5:54	CW004391.D	Virginia L1	Rep 1	51	51	60	46	72
6/17/21 6:37	CW004392.D	Virginia L1	Rep 2	58	55	56	44	65
6/17/21 7:20	CW004393.D	Virginia L1	Rep 3	52	58	58	44	62
6/17/21 8:04	CW004394.D	Virginia L1	Rep 4	60	60	61	45	67
6/17/21 8:47	CW004395.D	Virginia L1	Rep 5	62	58	65	54	63
6/17/21 9:30	CW004396.D	Virginia L1	Rep 6	56	59	62	51	66
6/17/21 10:14	CW004397.D	Virginia L1	Rep 7	64	59	66	47	61
			StDev	5.0	3.1	3.9	3.7	3.9
		Virginia	**MDL**	15.6	9.6	12.2	11.7	12.1

MDL calibration curve for all CPs in Silt

The method detection limit (MDL) for the methylated versions of 2-CP, 2,4-DCP, 2,4,6-TCP, PCP and TCS was determined to be 16.0, 21.8, 15.0, 15.4 and 12.2 ng/mL (7 replicates using the single-tailed 99th percentile *t* statistic) respectively. The MDL was determined following the guidelines in EPA document EPA 821-R-16-006 "Definition and Procedure for the Determination of the Method Detection Limit, Revision 2," December, 2016. A linear 8-point calibration curve was generated for this study, covering a CP calibration range from 50 to 800 ng/mL.

				**2-CP**	**2,4-DCP**	**2,4,6-TCP**	**PCP**	**TCS**
Date Acquired	Data File Name	Sample Name	Misc Info	ng/mL	ng/mL	ng/mL	ng/mL	ng/mL
6/18/21 9:01	CW004404.D	Silt L1	Rep 1	64	71	68	61	71
6/18/21 9:44	CW004405.D	Silt L1	Rep 2	59	57	63	52	68
6/18/21 10:28	CW004406.D	Silt L1	Rep 3	54	54	57	49	65
6/18/21 11:11	CW004407.D	Silt L1	Rep 4	54	50	58	46	66
6/18/21 11:55	CW004408.D	Silt L1	Rep 5	51	54	58	49	59
6/18/21 12:38	CW004409.D	Silt L1	Rep 6	52	50	54	49	63
6/18/21 13:22	CW004410.D	Silt L1	Rep 7	49	54	56	51	62
			StDev	5.1	6.9	4.8	4.9	3.9
		Silt	**MDL**	16.0	21.8	15.0	15.4	12.2
